# OE-YOLO: An EfficientNet-Based YOLO Network for Rice Panicle Detection

**DOI:** 10.3390/plants14091370

**Published:** 2025-04-30

**Authors:** Hongqing Wu, Maoxue Guan, Jiannan Chen, Yue Pan, Jiayu Zheng, Zichen Jin, Hai Li, Suiyan Tan

**Affiliations:** 1 College of Electronic Engineering, South China Agricultural University, Guangzhou 510642, China; 13665272593@163.com (H.W.); 19865586553@163.com (M.G.); chenjiannan@stu.scau.edu.cn (J.C.); yuki.pan2023@outlook.com (Y.P.); 15219487450@163.com (J.Z.); 19103498803@163.com (Z.J.); 2 Center for Basic Experiments and Practical Training, South China Agricultural University, Guangzhou 510642, China

**Keywords:** YOLOv11, oriented bounding boxes (OBB), EfficientNetV2, dynamic convolution, rice panicle, object detection, precision agriculture

## Abstract

Accurately detecting rice panicles in complex field environments remains challenging due to their small size, dense distribution, diverse growth directions, and easy confusion with the background. To accurately detect rice panicles, this study proposes OE-YOLO, an enhanced framework derived from YOLOv11, incorporating three synergistic innovations. First, oriented bounding boxes (OBB) replace horizontal bounding boxes (HBB) to precisely capture features of rice panicles across different heights and growth stages. Second, the backbone network is redesigned with EfficientNetV2, leveraging its compound scaling strategy to balance multi-scale feature extraction and computational efficiency. Third, a C3k2_DConv module improved by dynamic convolution is introduced, enabling input-adaptive kernel fusion to amplify discriminative features while suppressing background interference. Extensive experiments on rice Unmanned Aerial Vehicle (UAV) imagery demonstrate OE-YOLO’s superiority, achieving 86.9% mAP50 and surpassing YOLOv8-obb and YOLOv11 by 2.8% and 8.3%, respectively, with only 2.45 M parameters and 4.8 GFLOPs. The model has also been validated at flight heights of 3 m and 10 m and during the heading and filling stages, achieving mAP50 improvements of 8.3%, 6.9%, 6.7%, and 16.6% compared to YOLOv11, respectively, demonstrating the generalization capability of the model. These advancements demonstrated OE-YOLO as a computationally frugal yet highly accurate solution for real-time crop monitoring, addressing critical needs in precision agriculture for robust, oriented detection under resource constraints.

## 1. Introduction

Rice is crucial for global food security, especially in Asia and Africa, where over 3.5 billion people depend on it. Its growing areas cover 28% of the world’s arable land, with annual production exceeding 750 million tons [1]. As climate change and population growth intensify, improving rice yields through precision field management has become a key agricultural priority. Accurate panicle detection and counting have an irreplaceable role to play in increasing rice production and scientific field management. Not only can it help farmers rationally plan planting density, precisely apply fertilizer, improve nutrient utilization efficiency, and then improve rice yield [2], but it is also a key step in rice yield prediction. At the same time, real-time monitoring of the growth of rice panicles can help timely detect pests and diseases, providing a basis for taking effective control measures to reduce crop losses [3].

First, machine learning frameworks emerged as a sophisticated solution in rice panicle detection. Lingfen Duan et al. [4] employed artificial neural networks (ANN) to automate feature learning, offering adaptive capabilities beyond rule-based systems. However, this transformation brought new trade-offs: reduced interpretability, increased computational demands, and vulnerability to data biases.

Faced with these limitations of traditional machine learning, deep learning provides a breakthrough solution for rice panicle detection through its automated feature extraction and hierarchical pattern recognition capabilities. It demonstrates unparalleled robustness in complex field environments compared to traditional methods, effectively mitigating the sensitivity of manual feature design to data biases. Chaurasia et al. [5] and Suiyan Tan et al. [6] have advanced panicle detection through deep learning, yet their data acquisition approaches face distinct limitations. PanicleDet relies on controlled greenhouse imagery constrained by licensing restrictions and limited field applicability. Moreover, RiceRes2Net’s smartphone-based collection introduces challenges like narrow field-of-view and environmental noise. These factors collectively constrain model generalization across diverse agricultural scenarios.

To meet the escalating demands for precise rice panicle detection in modern agriculture, recent research advancements have focused on integrating deep learning with innovative data acquisition strategies. A key breakthrough lies in leveraging unmanned aerial vehicles (UAVs) to collect high-resolution RGB imagery, complemented by standardized horizontal bounding box (HBB) annotations. This approach systematically captures panicle morphological traits across diverse growth stages and environmental conditions while maintaining annotation consistency critical for model generalization. Chengquan Zhou et al. [7] pioneered this framework by training an improved R-FCN network on UAV-acquired images annotated with horizontal bounding boxes, effectively addressing panicle edge variations through data augmentation. Building upon this foundation, Maoyang Lan et al. [8] further optimized detection performance using YOLOv5 enhanced with EMA attention and SIoU loss, demonstrating the robustness of HBB-annotated UAV datasets under complex field scenarios. Concurrently, Jingwei Sun et al. [9] developed RP-YOLO for real-time rice panicle detection in unmanned harvesting systems, leveraging HBB-annotated japonica rice datasets and architectural modifications to achieve high-efficiency localization. These studies highlight how to integrate UAV technology with axis-aligned bounding box annotations. However, HBB annotations introduce geometric constraints that compromise detection accuracy. Traditional bounding box methods fail to adapt to natural panicle orientations and dynamic field conditions, leading to reduced accuracy for tilted targets and increased false positives in dense clusters. These limitations stem from horizontal bounding boxes’ inability to align with complex growth patterns under UAV imaging.

To address these challenges, oriented bounding boxes (OBB) have emerged as a promising alternative. Yunting Liang et al. [10] developed a YOLOv5-based model for rice phenotyping, incorporating circular smooth label angle classification and attention mechanisms to align bounding boxes with natural spike orientations, thereby reducing overlapping errors in dense clusters. Concurrently, Zhiheng Lu et al. [11] applied similar OBB principles to post-harvest sugarcane processing, integrating SE attention and CSL encoding to detect occluded plant segments. Can Wang et al. [12] proposed an Oriented RCNN-based framework for greenhouse detection in remote sensing imagery, incorporating PKINet with multi-scale convolution kernels to address scale variations. The method predicts rotation angles to align OBB precisely with greenhouse orientations, addressing dense distribution and directional challenges through hierarchical feature fusion and adaptive loss weighting. By enabling precise alignment with object geometry and reducing background noise, OBB-based methods represent a significant advancement in agricultural computer vision.

Although the aforementioned studies have made some progress in rice panicles, the existing methods still face multiple challenges in practical applications. First, due to complex phenotypic variations across growth stages and the complexity of field environment, the current algorithms demonstrate significant limitations in adapting to multiple growth stages and complex backgrounds. This primarily stems from insufficient feature extraction capabilities for panicle morphological diversity and environmental interference. Second, while most existing research focuses on accuracy improvement, the neglect of algorithm lightweight design results in inadequate computational efficiency when processing high-resolution field images, making it difficult to support real-time monitoring requirements. More critically, when panicles exhibit mutual occlusion or overlapping distributions, current approaches frequently suffer from feature confusion, causing missed or false detections—a critical bottleneck hindering practical implementation of these technologies.

In view of the problems in detection accuracy, speed, and adaptability of current methods, this research innovatively conducted large-scale data collection and achieved precise detection of dense rice panicles in the fields. By using the rice panicle images captured by UAVs at different growth stages and heights, a highly representative dataset was constructed. Subsequently, an improved YOLOv11, the OE-YOLO (Oriented–EfficientNetV2-YOLOv11) network, is proposed. In this network, the OBB (Oriented Bounding Boxes) model was applied instead of HBB (Horizontal Bounding Boxes) to improve the detection accuracy and reliability. The backbone network of YOLOv11 was replaced with EfficientNetV2, and by leveraging its powerful feature-extraction capabilities, the problem of low recognition efficiency of existing models for rice panicle features was effectively solved. The dynamic convolution mechanism was also introduced to form C3k2_DConv, enhancing the adaptability of the model in complex field environments. After the model improvement, comparative experiments were conducted on the improved models and the traditional models on the same dataset. Ablation study was also carried out to verify the discrimination ability of the adopted modules for dense rice panicles. Finally, a scientific evaluation system including mean average precision (mAP) and recall were adopted to comprehensively assess the performance of the improved model in the task of counting dense rice panicles in the fields. Through these innovations, OE-YOLO addresses the limitations of traditional deep learning in rice panicle detection. The architecture achieves a state-of-the-art performance across the different rice growth stages, significantly outperforming the existing methods. This provides an advanced, reliable, and high-performance solution for agricultural production, contributing to the new stage of precision agriculture development.

## 2. Materials and Methods

### 2.1. Experimental Locations

**Figure 1:**
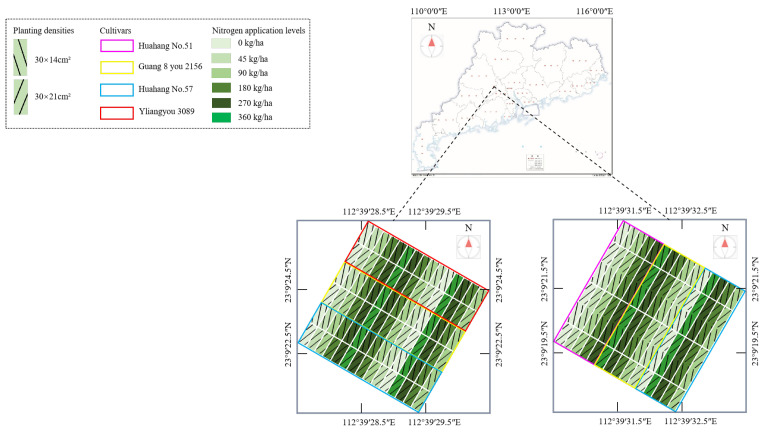
The research site.

The image was captured at the Sha Pu Research Center in Zhaoqing City, Guangdong Province (23.16° N, 112.66° E), China, as shown in Figure 1. Zhaoqing City is characterized by a subtropical monsoon climate, with a warm temperature throughout the year, aver-aging 21.8 °C annually. The area receives abundant precipitation with an annual rainfall of approximately 1652.6 mm and experiences around 1672.8 h of sunlight annually. Overall, the climate of Zhaoqing is warm and humid, making it suitable for agricultural production, especially rice cultivation. A double-season rice planting scheme was implemented in spring and autumn. The experimental field adopts individual plot planting strategies, where each plot follows a different planting configuration. The configurations include three rice varieties, five nitrogen fertilizer levels, and two trans-planting densities, resulting in a total of 30 planting modes. Each planting mode is repeated three times, leading to a total of 90 experimental plots per growing season. Among them, the extreme nitrogen fertilizer treatment intentionally enhances the phenotypic diversity of rice growth, thereby rigorously validating the model’s robustness to morphological extremes and laying a critical foundation for yield prediction under heterogeneous field conditions. The area of each plot is 10.8 × 3.5 m^2^.

**Figure 2:**
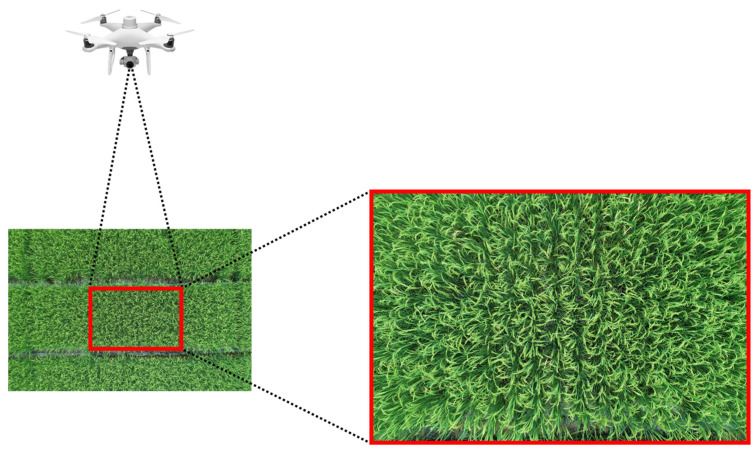
UAV Captured Image.

To ensure image diversity, rice panicle images under different planting conditions and on different dates were captured using a DJI Phantom 4 RTK UAV (Shenzhen, China). The primary method involved controlling the UAV to fly over the center of each plot for image acquisition, with the shooting process illustrated in Figure 2. The images were taken vertically, with 3–4 images selected per plot. The flight heights were 3 m and 10 m, primarily to minimize the impact of wind generated by the UAV’s wings on the rice panicle, and also adopted to compare detection performance of deep learning models at different heights.

After several visits to the experimental fields, the data and images were collected during the spring and autumn seasons in 2021 and 2024, respectively. The large time span of data collection is mainly due to the long planting cycle of rice and the differences in data quality each year; the data from 2021 and 2024 already cover the basic information of the paddy fields and are sufficient for conducting this research. The experimental data in spring (EXP.1) and autumn (EXP.2) consist of original rice panicle RGB images and were used to construct the comprehensive dataset. Subsequent data for model training were selected from this dataset and then subjected to data preprocessing. The specific data are shown in Table 1.

### 2.2. Image Preprocessing

**Figure 3:**
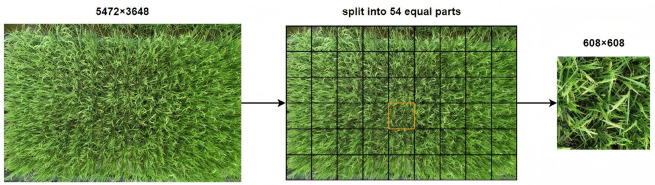
Equal partitioning of images.

The original images collected by UAV have a resolution of 5472 × 3648. Directly inputting them into the deep learning models would lead to a loss of resolution and the potential loss of some features. Therefore, the images were cropped first by using a Python program, divided into 54 equal parts, resulting in a resolution of 608 × 608 pixels. A diagram illustrating the image cropping process is shown in Figure 3. After the cropping, RolabelImg was used to annotate the rice panicles in the images. This annotation method helps reduce the influence of background factors on the detection process.

**Figure 4:**
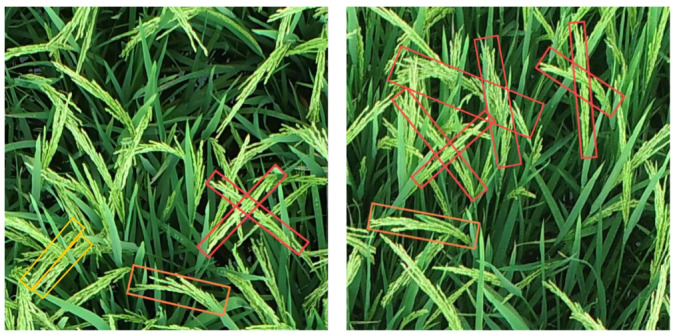
Rice panicle OBB annotation instances. The red, orange, and yellow rectangles represent the crossed, curved, and parallel rice panicles, respectively.

The RolabelImg tool was adopted to conduct oriented bounding box annotations on images, which were stored in XML files containing five parameters, including object center coordinates (cx, cy), width (w), height (h), and rotation angle (θ). The complex field environment poses significant challenges for accurate rice panicles annotation. To ensure consistency and reliability during dataset construction, the following annotation protocols were established. First, for an individual panicle without occlusion, align the rotation angle θ with the main stem of the rice panicle and bounding boxes to enclose all its visible parts. Second, in scenarios of partial occlusion where more than 30% of the panicle is visible, only the visible part is annotated. If a visible portion of the panicle is less than 30%, OBB should not be included. Third, for curved rice panicles, OBB’s θ should be aligned with the curvature of the curve and bounding boxes to enclose all visible parts of the panicle. In order to visualize these protocols, Figure 4 clearly illustrates examples of annotations under different conditions, such as crossed, curved, and parallel arrangements of rice panicles.

**Figure 5:**
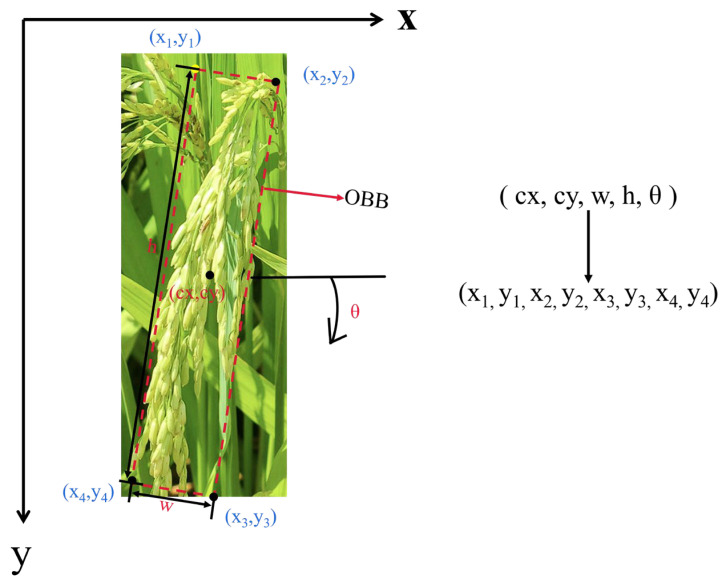
Visualization of the format conversion of annotations.

Since YOLOv11 cannot directly process XML format, a two-step annotation format conversion is performed, as shown in Figure 5. The RolabelImg XML files are first converted to the DOTA remote sensing dataset-compatible TXT format, and then further transformed into TXT files trainable by YOLOv11, including four vertex coordinates of oriented bounding boxes (x1, y1, x2, y2, x3, y3, x4, y4), ensuring compatibility with the model’s orientation-aware training requirements.

**Figure 6:**
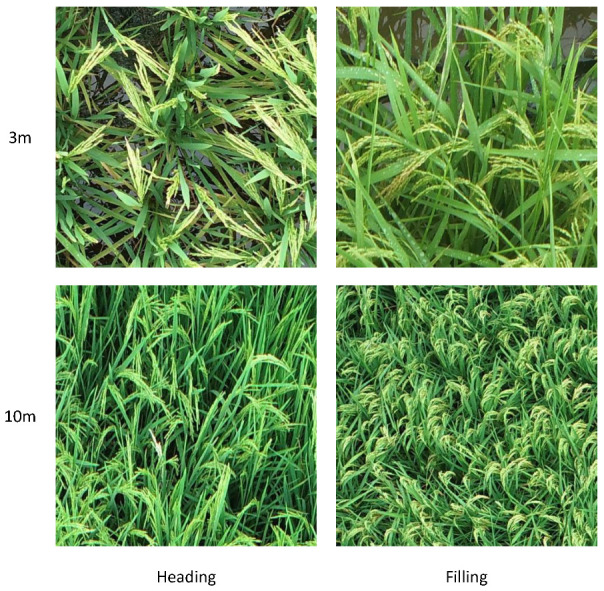
Rice panicle images at different heights and growth stages.

**Figure 7:**
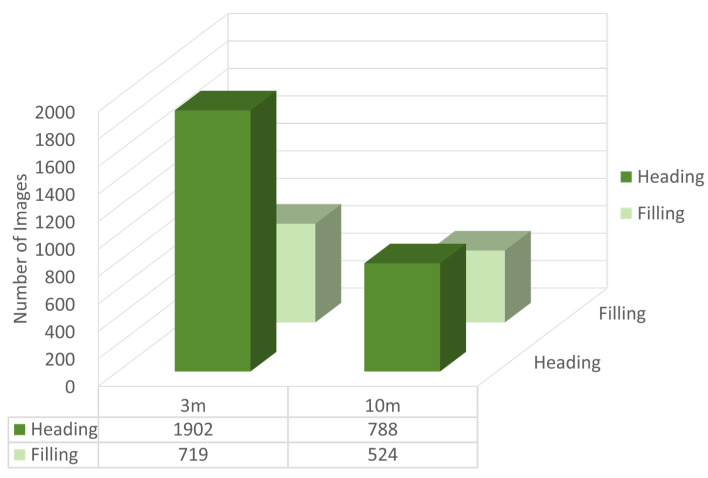
Dataset distribution.

After image cropping and annotation, a total of 1902 images of rice panicles at the heading stage and 719 images at the filling stage were acquired under 3 m conditions, along with 788 images of rice panicles at the heading stage and 524 images at the filling stage under 10 m conditions. The rice panicle images acquired at different flight heights and growth stages are shown in Figure 6, and the dataset distribution are displayed at Figure 7. In addition, appropriate augmentation of the dataset can enhance the model’s generalization ability and reduce the occurrence of overfitting during model training; therefore, data augmentation was performed, including horizontal flipping, affine transformations (scaling, translation, rotation), contrast adjustment, and Gaussian blur.

### 2.3. Methods

#### 2.3.1. YOLOv11 Networks

YOLOv11 was developed by the Ultralytics research team in September 2024, representing the latest advancement in real-time object detection algorithms [13]. This iteration introduces significant improvements in speed, accuracy, and adaptability, marking a notable leap forward from its predecessors. Its key innovations include the C3k2 mechanism and the C2PSA (Cross-Stage Partial with Self-Attention) module, which enhance the model’s feature extraction and representation capabilities.

The C3k2 mechanism is an optimized variant of the C2f module, which employs two smaller convolutional operations instead of a single large convolutional layer [14]. This approach balances accuracy, efficiency, and processing speed, reducing both the number of parameters and computational load while maintaining high detection precision. Such improvements make the model particularly suitable for real-time applications.

Another critical advancement is the integration of the C2PSA module within the backbone architecture. This module combines cross-stage partial networks with self-attention mechanisms, enabling the model to capture contextual information across multiple layers effectively. The self-attention mechanism allows the model to focus on crucial regions of the image, enhancing detection accuracy and efficiency, especially for small or occluded objects.

**Figure 8:**
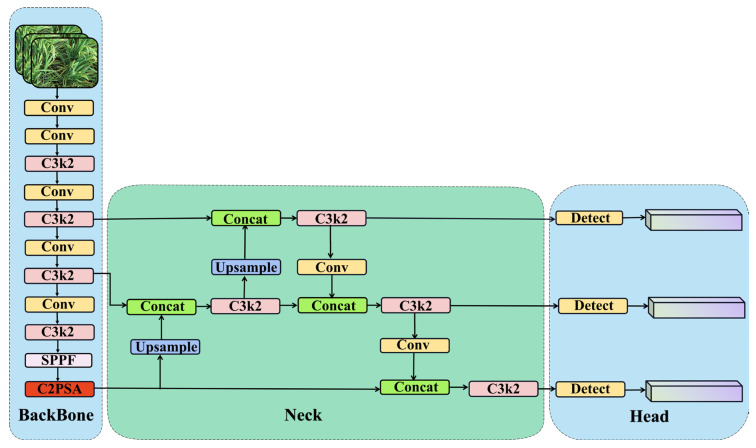
Network structure of YOLOv11.

The backbone architecture of YOLOv11, illustrated in Figure 8, consists of multiple convolutional layers and C3k2 modules, followed by concatenation and detection layers. This structure enhances the model’s ability to handle complex tasks through more precise and effective feature extraction. The detection head has been optimized by replacing DWConv, further reducing computational load while maintaining high detection precision.

#### 2.3.2. OE-YOLO Networks Construction

YOLOv11, while being a notable object-detection framework, exhibits certain drawbacks when applied to rice panicle detection. Primarily, the use of HBB restricts its ability to precisely localize rice panicles, as rice panicles in the field often have irregular shapes and orientations, leading to the inclusion of more background and less accurate detections. Additionally, its backbone network consumes excessive computational resources. The conventional C3k2 modules in YOLOv11 also lack adaptability to the diverse features of rice panicles in different scenarios.

**Figure 9:**
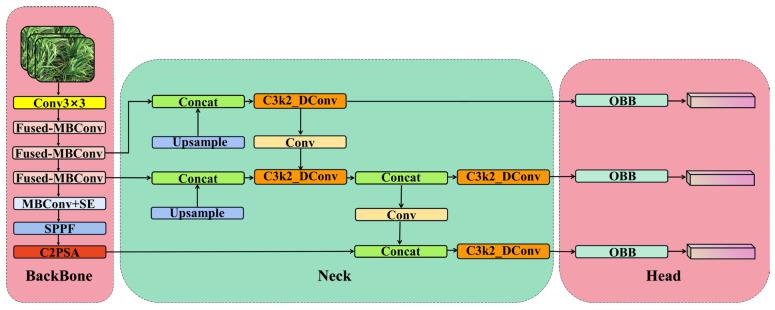
Network structure of OE-YOLO.

Therefore, the proposed OE-YOLO network addresses these limitations as shown in Figure 9. The first significant enhancement is the implementation of OBB. In the OE-YOLO structure, the OBB model in the head part replaces the traditional HBB. This change allows OE-YOLO to better conform to the shapes and orientations of rice panicles. By using OBB, the model can more accurately enclose the rice panicles, minimizing the inclusion of background elements and significantly improving the precision of object localization, especially in dense rice-field environments where accurate object separation is vital.

The second key improvement is the substitution of the YOLOv11 backbone with the lightweight EfficientNetV2. EfficientNetV2 is chosen for its remarkable balance between computational efficiency and feature extraction capability. It can effectively extract hierarchical features from rice images, encompassing both the overall context and local characteristics of rice panicles. Its lightweight nature ensures that the model remains computationally feasible for real-world agricultural applications with limited resources, maintaining detection accuracy while reducing the computational burden compared to the original backbone.

The third improvement lies in the integration of Dynamic Convolution into C3k2 to form C3k2_DConv. In the neck section of the OE-YOLO structure, these C3k2_DConv modules are strategically placed. Dynamic Convolution endows the C3k2 modules with the ability to adapt to different input features. This adaptability enables the model to better capture the context-specific and variable features of rice panicles in various growth stages and environmental conditions.

#### 2.3.3. OBB

While the conventional YOLOv11 model employs HBB for object containment, this study introduces an OBB model with angular positioning descriptors to address the challenges posed by the slender shape of rice panicles and the cluttered agricultural background [13]. The OBB approach demonstrates superior performance over HBB in suppressing background interference and improving alignment accuracy with inclined rice panicles under complex field conditions.

**Figure 10:**
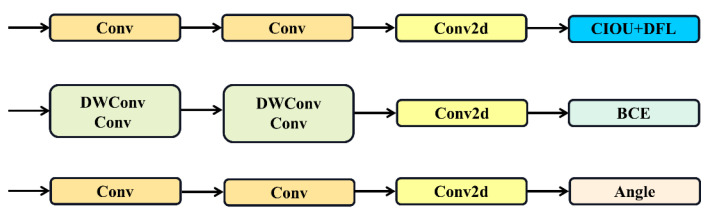
Architecture of OBB in YOLOv11.

The proposed OBB architecture has made specific improvements to the detection head. Compared with the original version, the OBB model incorporates an angular parameter prediction module that predicts the orientation information (angle) of oriented bounding boxes through a newly added angular branch. This angular parameter represents the rotation angle relative to the target center, as depicted in Figure 10.

For the loss function design, the OBB model replaces the conventional Complete Intersection-over-Union (CIoU) with a Probabilistic Intersection-over-Union (ProbIoU), which is based on probability distribution modeling [15]. According to the derivation, ProbIoU measures bounding box overlap through a similarity assessment between Gaussian distributions. Given the parameters of the two-dimensional Gaussian distribution (mean μ, covariance matrix Σ) for the predicted and ground truth bounding boxes, the core calculation is shown in Equations (1)–(3), expressed as

(1) $$ProbIoU=1-H_{D}\left(p,q\right)$$

(2) $$H_{D}\left(p,q\right)=\sqrt{1-B_{C}\left(p,q\right)}$$

(3) $$B_{C}(p,q)=\int_{R^{2}}\sqrt{p(x)q(x)}dx.$$

Here, ProbIoU represents the probabilistic intersection-over-union based on the Hellinger Distance, which is used to measure the overlap degree between two Gaussian distributions. $H_{D}$ denotes the Hellinger Distance between the two Gaussian distributions p and q, which measures the difference between two probability distributions. $B_{C}$ represents the Bhattacharyya Coefficient between the two probability density functions p and q.

Compared to CIoU, ProbIoU offers three distinct advantages: rotation invariance, gradient stability, and geometric adaptability. Rotation invariance is achieved by naturally encoding directional information through the covariance matrix, eliminating the need for explicit angular constraints. Gradient stability is ensured as the probabilistic overlap measure that maintains effective gradient propagation even in non-overlapping scenarios. The elliptical fitting of ProbIoU more closely approximates the true shape of the target, thereby reducing mismatches with background regions.

#### 2.3.4. EfficientNetV2

In the domain of object detection, YOLOv11 has been widely adopted for its unique strengths, yet its original backbone network exhibits notable limitations in practical applications. Primarily, the architecture struggles with inefficient training when processing high-resolution images due to excessive memory consumption, which forces the use of small batch sizes and consequently slows training convergence. Additionally, the inefficient implementation of depthwise convolutions in early layers fails to fully leverage modern hardware accelerators. The uniform scaling strategy across network stages further leads to suboptimal parameter utilization, potentially undermining feature extraction capabilities and detection performance.

**Figure 11:**
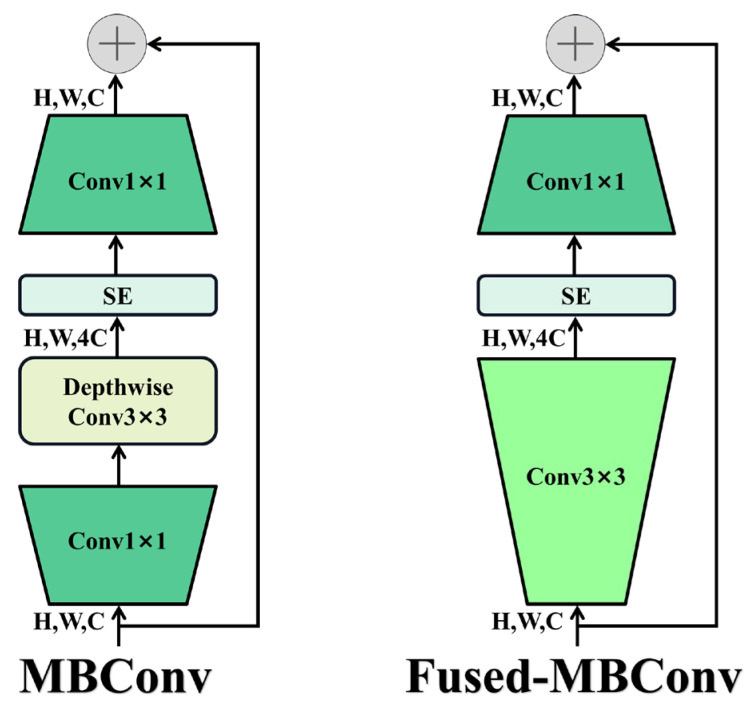
Structure of the MBConv and Fused-MBConv.

In contrast, EfficientNetV2 addresses these challenges through a training-aware neural architecture search and scaling methodology, jointly optimizing convolutional operator types, layer counts, kernel sizes, and expansion ratios. This approach yields a more computationally efficient and feature-rich architecture. Its structural parameters are shown in Table 2. Central to its design are the MBConv and Fused-MBConv modules [16]. The MBConv module employs a 1 × 1 convolution for channel expansion, followed by a 3 × 3 depthwise convolution for spatial feature extraction, and a 1 × 1 convolution for channel reduction [17]. This structure balances parameter efficiency with robust feature representation. The Fused-MBConv variant replaces the depthwise and expansion convolutions in MBConv with a single 3 × 3 standard convolution, enhancing compatibility with hardware accelerators in shallow layers and significantly accelerating training (Figure 11) [18].

EfficientNetV2-S further optimizes architectural parameters by adopting smaller expansion ratios and 3 × 3 kernel sizes, compensating for reduced receptive fields through increased layer depth. It eliminates the final stride-1 stage from the original EfficientNetV2 architecture, thereby reducing parameter counts and memory access overhead [19]. Complementing these structural enhancements is an improved progressive learning strategy: training begins with smaller image dimensions and weaker regularization to rapidly capture low-level features, then gradually scales image sizes and intensifies regularization to refine high-level representations. This approach mitigates accuracy loss while strengthening feature discriminability and generalization.

To integrate EfficientNetV2-S into YOLOv11, critical components are retained and adapted. Convolution, batch normalization, and activation functions are combined to form the foundational unit for feature extraction. The SE module incorporates squeeze-and-excitation mechanisms to adaptively recalibrate channel-wise feature responses, emphasizing task-critical information. Stochastic depth regularization is applied during training, randomly bypassing specific layers to enhance robustness and mitigate overfitting.

EfficientNetV2-S demonstrates superior training efficiency and detection accuracy in rice panicle detection tasks. Its optimized MBConv and Fused-MBConv modules minimize parameter redundancy and computational costs, enabling rapid training on large-scale rice image datasets under constrained resources. The architecture’s hierarchical feature extraction excels at capturing fine-grained details, such as panicle morphology and growth-stage characteristics, directly improving detection precision and recall. By harmonizing computational efficiency with representational power, EfficientNetV2-S emerges as an excellent backbone for advancing YOLOv11-based rice panicle detection systems.

#### 2.3.5. C3k2_DConv: Integration of Dynamic Convolution into C3k2

Rice panicle detection presents unique challenges due to the crop’s slender morphology, dense spatial distribution, and complex growth environments. Rice panicles often occupy small regions in images, with limited pixel information and discriminative features, while their dense arrangement leads to frequent occlusion and overlap. Furthermore, background elements such as soil and weeds exhibit visual similarities to rice in color, texture, and shape, exacerbating confusion during detection [20].

The original C3k2 module in YOLOv11 struggles to address these challenges effectively. Its reliance on fixed convolutional kernels limits adaptability to the diversity and complexity of rice panicles. For instance, subtle features like slender stems and tender leaves during the heading stage are often inadequately captured, degrading detection accuracy. In dense scenarios, fixed receptive fields fail to adapt to overlapping instances, resulting in missed detections or false positives. Additionally, static convolution operations cannot dynamically prioritize foreground features over complex backgrounds, leading to erroneous identifications of non-target elements.

**Figure 12:**
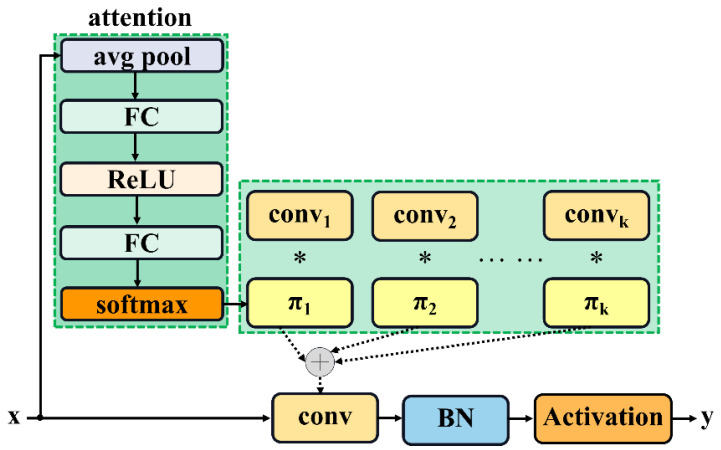
Dynamic perceptron. It dynamically aggregates multiple linear functions based on the input attentions {$\pi_{k}$}.

To mitigate these limitations, we integrate Dynamic Convolution into the C3k2 module, forming the enhanced C3k2_DConv structure. Dynamic Convolution enhances feature extraction by adaptively aggregating multiple convolutional kernels based on input characteristics [21]. Let $\{{\tilde{W}}_{k}{\}}_{k=1}^{K}$ parallel convolutional kernels. For an input feature map x, attention weights $\{\pi_{k}(x){\}}_{k=1}^{K}$ are computed to dynamically fuse these kernels (Figure 12) [22]. The calculation is shown in Equation (4):

(4) $$\tilde{W}\left(x\right)=\sum_{k=1}^{K}\;\pi_{k}\left(x\right){\tilde{W}}_{k}\;where\;0\leq \pi_{k}\left(x\right)\leq 1\;and\;\sum_{k=1}^{K}\;\pi_{k}\left(x\right)=1.$$

Similarly, the bias term $\tilde{b}\left(x\right)$ is aggregated as Equation (5):

(5) $$\tilde{b}\left(x\right)=\sum_{k=1}^{K}\;\pi_{k}\left(x\right){\tilde{b}}_{k}.$$

This mechanism allows the model to tailor convolutional operations to input-specific features, enhancing adaptability.

The attention weights $\pi_{k}\left(x\right)$ are derived through a gating network. First, as shown in Equation (6), global average pooling condenses the input feature map $x\in R^{C\times H\times W}$ into a channel-wise vector:

(6) $$\mathrm{pooled}\_\mathrm{inputs}=\frac{1}{H\times W}\sum_{i=1}^{H}\;\sum_{j=1}^{W}\;x\left[:,i,j\right].$$

This vector is processed by a multi-layer perceptron (MLP) with weights $W_{1}\in R^{C_{1}\times C},\;W_{2}\in R^{K\times C_{1}}$ and biases $b_{1}\in R^{C_{1}},\;b_{2}\in R^{K}$. The calculation is shown in Equations (7) and (8):

(7) $$h=ReLU\left(W_{1}\cdot \mathrm{pooled}\_\mathrm{inputs}+b_{1}\right),$$

(8) $$z=W_{2}\cdot h+b_{2}$$

where *h* is the hidden layer output and $Z\in R^{K}$. Finally, the attention weights are normalized via softmax in Equation (9):

(9) $$\pi_{k}\left(x\right)=\frac{e^{z_{k}}}{\sum_{j=1}^{K}\;e^{z_{j}}}.$$

This input-dependent weighting enables the model to emphasize task-relevant features, such as rice panicles, while suppressing background interference.

In the C3k2_DConv architecture, the original C3k2 framework is retained but augmented with Dynamic Convolution. Initial feature extraction is performed by the baseline C3k2 layers, followed by dynamic refinement in the bottleneck stage. Let $x_{1}=Conv(x)$ denote the output of the initial convolutional layer. The dynamic convolution operation is then applied in Equation (10):

(10) $$x_{2}=\sum_{k=1}^{K}\;\pi_{k}\left(x_{1}\right){\tilde{W}}_{k}*x_{1},$$

where * denotes convolution. This adaptive mechanism allows the model to adjust receptive fields and kernel weights based on localized features—enhancing sensitivity to small rice targets, resolving occlusions in dense clusters, and mitigating background confusion.

By synergizing the structural robustness of C3k2 with the adaptability of Dynamic Convolution, C3k2_DConv achieves superior performance in rice panicle detection. For immature seedlings during the heading stage, it prioritizes kernels that amplify fine details; for mature rice during the filling stage, it emphasizes structural patterns. This flexibility ensures precise feature extraction across growth stages and environmental conditions, significantly improving detection accuracy and reliability in agricultural applications.

## 3. Results

### 3.1. Experimental Setup

#### 3.1.1. Experimental Platform

The experiments were conducted on a computational platform comprising an Intel Core i9-10700KF CPU and an NVIDIA GeForce RTX 3090 GPU to ensure high-performance parallel processing (LENOVO, Beijing, China). The software environment utilized Windows 11 as the operating system, with PyCharm serving as the integrated development environment (IDE) and Python 3.8 for algorithmic implementation. Model training spanned 110 epochs, initialized with a learning rate of 0.01 and dynamically adjusted via a cosine annealing strategy to enhance convergence stability. Input images were standardized to a resolution of 608 × 608 pixels, aligning with the training sample dimensions. To mitigate overfitting, an L2 regularization term with a weight decay coefficient of 0.001 was integrated into the optimization framework. This configuration balanced computational efficiency and model generalization across the experimental pipeline.

#### 3.1.2. Evaluation Indicators

To comprehensively evaluate the performance and generalization capability of the proposed model, a set of evaluation metrics were employed to assess different aspects of its effectiveness. Precision (P) and recall (R), as shown in Equations (11) and (12), provide insights into the accuracy and robustness of feature extraction under varying conditions or noisy datasets. To be specific, macro-average is adopted for P and R calculations to ensure equitable evaluation across all instances, preventing dominance by large or densely clustered targets while sensitively capturing performance variations for small or partially occluded objects. The average precision (AP) is computed by integrating the area under the precision-recall curve with all-point interpolation (Equation (13)). The mAP, as shown in Equation (14), further extends these evaluations by considering performance across multiple queries or tasks, ensuring the model’s reliability in real-world applications [23]. To rigorously evaluate detection performance across varying localization strictness while balancing sensitivity to small targets and tolerance for inherent field positioning errors, both mAP50 (IoU threshold = 0.5) and mAP50-95 (mean average precision averaged over IoU thresholds from 0.5 to 0.95 in 0.05 increments) are adopted. The definition of IoU is shown in Equation (15), where A and B represent the predicted bounding box and the ground-truth bounding box, respectively. Anchors with an IoU ≥ 0.7 relative to ground-truth boxes were assigned as positive samples, ensuring precise localization for slender rice panicles during training. Additionally, parameters and Giga Floating Point Operations (GFLOPs) are calculated to quantify the computational demands during training, offering insights into scalability and suitability for resource-constrained environments [24]. These metrics collectively ensure a thorough assessment of both accuracy and practicality in model’s performance evaluation:

(11) $$P=\frac{TP}{TP+FP}$$

(12) $$R=\frac{TP}{TP+FN}$$

(13) $$AP=\int_{0}^{1}\;PdR$$

(14) $$mAP=\frac{1}{N}\sum_{j=1}^{N}\;AP_{j}$$

(15) $$IoU=\frac{A\cap B}{A\cup B},$$

where TP signifies the quantity of samples that are accurately classified as positive; FP signifies the number of samples misclassified as positive; and FN signifies the number of samples wrongly classified as negative [25].

### 3.2. Experimental Result

#### 3.2.1. Ablation Studies

**Figure 13:**
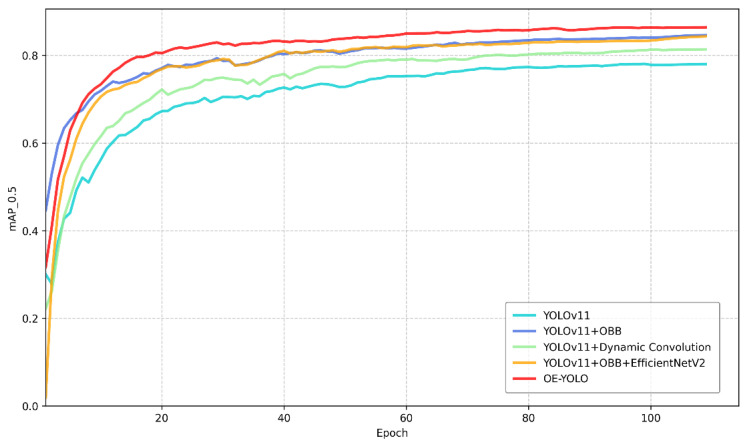
Comparison of mAP50 for each model.

In the ablation experiments of the study using OE-YOLO to detect rice panicles, the contributions of each improved component were systematically assessed, and the results are shown in Table 3. To gain a more intuitive understanding of the performance of each model in terms of mAP, Figure 13 is presented.

First, when only YOLOv11n was used without any additional improvements, mAP50 was 78.6%, with 2.58 M parameters and 6.3 GFLOPs. Incorporating OBB alone led to a notable increase in mAP50 to 84.7%, and the number of parameters slightly rose to 2.65 M while the GFLOPs increased to 6.6. This indicates that OBB can effectively improve the recognition accuracy for rice panicles by better fitting their shapes.

Replacing the backbone network of YOLOv11 with EfficientNetV2 also brought positive results. The mAP50 was 78.8%, with a reduced parameter count of 2.09 M and a significant decrease in GFLOPs to 4.5. This shows that EfficientNetV2 can not only maintain relatively high accuracy but also improve the computational efficiency.

Integrating Dynamic Convolution into C3k2 to form C3k2_DConv improved the mAP50 to 81.2%, with 2.94 M parameters and 6.2 GFLOPs. This suggests that Dynamic Convolution can enhance the feature-extraction ability in the network for rice object recognition.

When combining OBB and EfficientNetV2, the mAP50 reached 84.5%, with 2.15 M parameters and 4.7 GFLOPs, demonstrating the complementary nature of these two improvements.

Finally, when all the improvements (OBB, EfficientNetV2 backbone replacement, and C3k2_DConv) were combined, OE-YOLO achieved the highest mAP50 of 86.9%, with 2.45 M parameters and 4.8 GFLOPs. This comprehensive improvement not only significantly enhanced the recognition accuracy but also maintained a relatively reasonable parameter and computational cost, indicating the effectiveness of the proposed improvement strategy for rice Panicle detection using OE-YOLO.

#### 3.2.2. Detection Effectiveness at Various Growth Stages and Flight Heights

**Figure 14:**
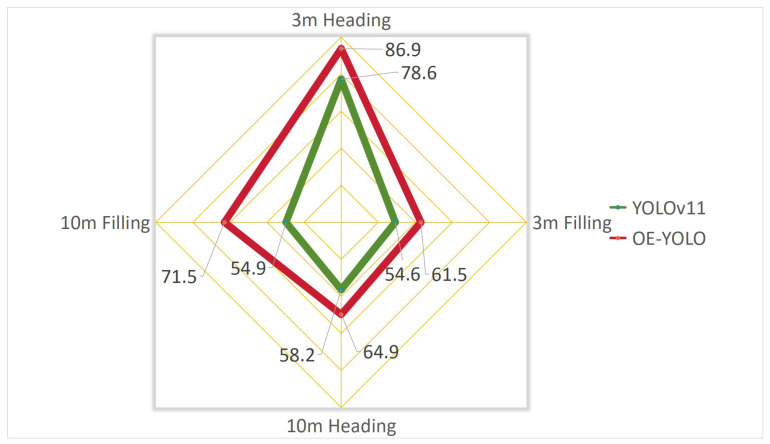
Comparison of mAP50 between YOLOv11 and OE-YOLO at different flight heights and growth stages.

The proposed OE-YOLO exhibits consistent performance improvements over the baseline YOLOv11 across diverse operational distances and growth stages (Table 4, Figure 14). Under 3 m normal lighting during the heading stage, OE-YOLO achieves a mAP50 of 86.9%, surpassing YOLOv11 (78.6%) by 8.3%, while simultaneously improving precision and recall by 10.9% and 10.1%, respectively. Notably, the improvements persist in challenging scenarios: at 10 m under filling-stage conditions, OE-YOLO attains a 16.6% higher mAP50, demonstrating enhanced robustness to scale variation and occlusion.

The performance disparity between 3 m and 10 m configurations underscores the impact of spatial resolution on detection fidelity. At 3 m, OE-YOLO’s mAP50 for heading-stage rice panicles reaches 86.9%, leveraging high-resolution imagery to resolve fine-grained features such as panicle orientation and stem curvature. This resolution advantage enables precise differentiation of slender panicles from cluttered backgrounds, particularly during the heading stage when morphological structures are well-defined. In contrast, 10 m scenarios suffer from resolution degradation, reducing mAP50 to 64.9% despite the model’s adaptive capabilities. The pixelation effects at elevated heights obscure critical morphological details (e.g., panicle-tip curvature), while overlapping instances and background elements (e.g., soil, weeds) exhibit heightened visual ambiguity under scale reduction—a challenge exacerbated during the filling stage when panicle density peaks. This trend is mirrored in precision-recall metrics: at 3 m flight heights, OE-YOLO achieves 83.9% precision and 82.8% recall for heading-stage detection, whereas 10 m precision declines to 57.9%, primarily due to false positives triggered by texture-similar background regions.

The 3 m heading-stage dataset was prioritized for subsequent experiments due to its optimal balance of annotation consistency, morphological richness, and practical relevance. From an operational perspective, the findings suggest a trade-off between coverage efficiency and detection accuracy: 3 m flight height is recommended for precision tasks like yield estimation, where panicle-level granularity is critical, while operations at 10 m flight height may suffice for large-scale phenotyping with tolerance for marginal accuracy loss. Heading-stage rice exhibits well-defined panicle structures with minimal occlusion, enabling precise OBB-based angle regression. Furthermore, 3 m heading-stage images provide sufficient spatial granularity to train dynamic convolution kernels for discriminative feature amplification, particularly for distinguishing panicle axes from inclined stems. However, the capability diminished at 10 m flight height due to resolution constraints.

#### 3.2.3. Experimental Analysis of EfficientNetV2 Backbone Selection

**Figure 15:**
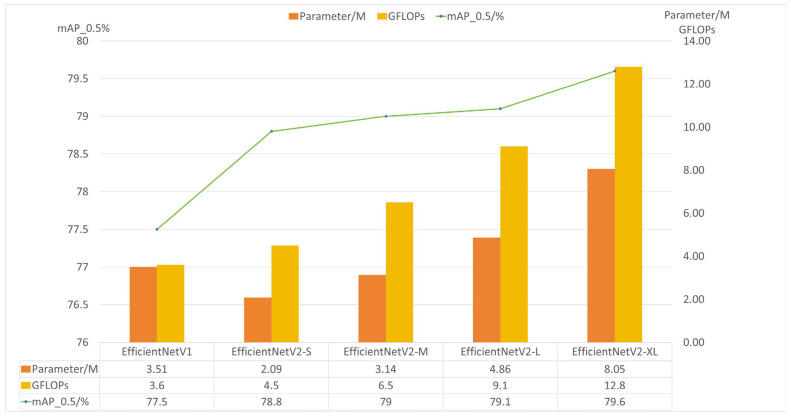
Comparison of the EfficientNet selection.

The architectural efficacy of EfficientNetV2 variants as backbone networks was systematically evaluated within the YOLOv11 framework for rice panicle detection. Comparative experiments were conducted across EfficientNetV1 and four scaled versions of EfficientNetV2 (S, M, L, XL), which are different from depth, width, and feature resolution [26], with performance metrics summarized in Figure 15. The results demonstrated a non-linear relationship between model complexity and detection accuracy. Specifically, EfficientNetV2-S achieved a mAP50 of 78.8% with only 2.09 M parameters and 4.5 GFLOPs, outperforming EfficientNetV1 (77.5% mAP50, 3.51 M parameters) by a margin of 1.3% while reducing parameter count by 40.7%. This highlights the effectiveness of architectural innovations in EfficientNetV2, including fused-MBConv layers and progressive learning strategies, in enhancing feature extraction efficiency.

Notably, the scalability analysis revealed diminishing returns with larger variants. While EfficientNetV2-XL attained the highest mAP50 of 79.6%, its computational cost (12.8 GFLOPs) and parameter size (8.05 M) increased by 184% and 285%, respectively, relative to EfficientNetV2-S, yielding merely a 0.8% accuracy gain. Such marginal improvements suggest limited practical value for resource-constrained agricultural applications. Furthermore, the computational efficiency metric (mAP50 per GFLOP) starkly favored EfficientNetV2-S (17.51) over EfficientNetV2-XL (6.22), reinforcing its suitability for real-time field deployment scenarios.

The selection of EfficientNetV2-S as the optimal backbone was further justified by its balanced trade-off between detection performance and operational feasibility. In rice panicle detection tasks, where intra-class variance due to growth stages and occlusion poses significant challenges, V2-S’s compact architecture effectively captured discriminative features without overfitting to the sparse training data. Ablation studies confirmed that deeper networks exhibited negligible accuracy improvements on small-scale rice datasets, likely due to redundant hierarchical representations. These findings align with the principle of model scaling in resource-aware computer vision, where excessive parameterization fails to proportionally enhance task-specific robustness.

This experiment underscores the criticality of backbone customization for crop detection systems. By adopting EfficientNetV2-S, the proposed model achieved state-of-the-art accuracy while maintaining deployability on edge devices, a prerequisite for scalable precision agriculture solutions.

#### 3.2.4. Experiment on C3k2_DConv Placement Strategies

To validate the effectiveness of the proposed C3k2_DConv module in multi-scale feature aggregation, the placement strategies were systematically evaluated across small (20 × 20), medium (40 × 40), and large (80 × 80) detection heads. As shown in Table 5, integrating C3k2_DConv into all three heads (Serial No. 8) achieves the highest mAP50 of 86.9%, surpassing configurations with partial placements (e.g., 85.5% for medium-head-only integration). Notably, the parameter count (2.45 M) and computational cost (4.8 GFLOPs) remain nearly stable across different configurations, with a marginal variation in less than 5% in both metrics. This suggests that the performance gain stems from enhanced feature representational capacity rather than parameter inflation.

**Figure 16:**
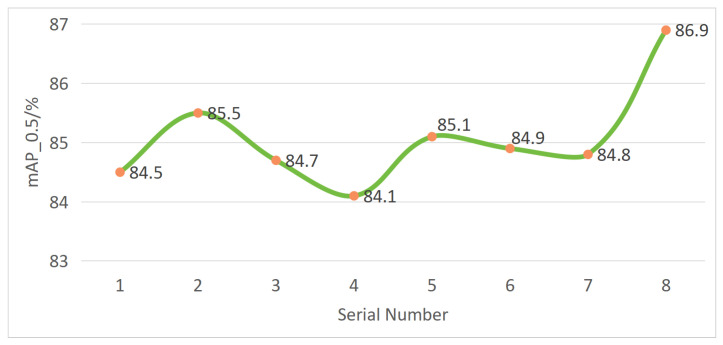
Visualization of mAP50 when C3k2_DConv module was placed in different positions.

The line chart in Figure 16 visualizes the progressive improvement in mAP50 more clearly as C3k2_DConv is incrementally deployed across scales. The full integration strategy (No. 8) exhibits a 2.4% absolute gain over the baseline (No. 1), highlighting the synergistic effect of dynamic convolution in addressing scale variance and background interference. Specifically, the adaptive kernel fusion in C3k2_DConv enables context-aware feature refinement: for small rice grains, it amplifies high-frequency details through spatially focused kernels; for densely clustered instances, it suppresses cross-boundary confusion via channel-wise attention modulation. Such adaptability is critical for rice panicle detection, where scale extremes and occlusion patterns coexist.

The minimal computational overhead (0.1 GFLOPs increment) further corroborates the architectural efficiency of our design. Unlike brute-force parameter stacking, the dynamic routing mechanism in C3k2_DConv selectively activates expert kernels based on input characteristics, ensuring computational resources are allocated to task-critical regions. This equilibrium between accuracy and efficiency positions our full-integration strategy as the better choice for real-world deployment in agricultural vision systems.

#### 3.2.5. Comparison of Different Models

The proposed OE-YOLO demonstrates significant advancements in rice panicle detection across accuracy, parameter efficiency, and computational cost compared to state-of-the-art models, as summarized in Table 6. OE-YOLO achieves a remarkable mAP50 of 86.9%, surpassing conventional detectors such as YOLOv5n (75.5%) and YOLOv8n (76.7%) by margins of 11.4% and 10.2%, respectively [27]. Notably, this performance also exceeds YOLOv8-obb (84.1%), validating the efficacy of integrating dynamic convolution and EfficientNetV2 for capturing rotational and fine-grained features in densely occluded rice fields. Furthermore, OE-YOLO outperforms specialized oriented detection frameworks, including Oriented R-CNN (80.8% mAP50), FCOSR (84.3% mAP50), and R3Det_tiny (81.1% mAP50), by significant margins [28,29,30]. While S^2^A-Net achieves the mAP50 of 85.2%, its 38.54 M parameter count and 196.2 GFLOPs computational demands render it impractical for real-time deployment [31].

In contrast, OE-YOLO maintains a lean parameter footprint of 2.45 M—only 5.9% of R3Det’s and 6.4% of S^2^A-Net’s. This efficiency stems from the synergy between EfficientNetV2’s compound scaling and C3k2_DConv’s adaptive kernel fusion, which minimizes redundant computations without sacrificing discriminative power.

**Figure 17:**
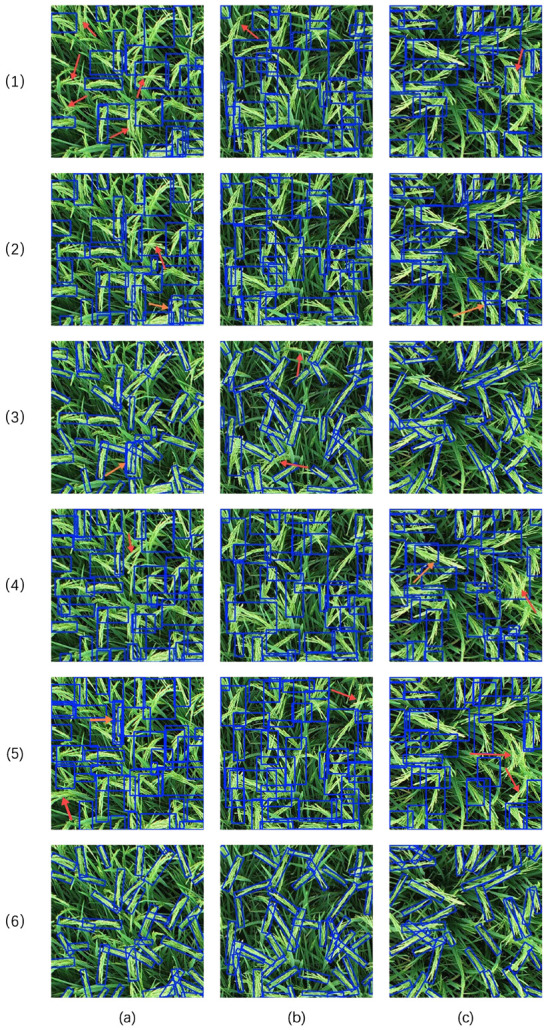
Comparison of detection results of different models. The blue rectangular boxes represent the predicted targets. Three different rice panicle images (**a**–**c**) were randomly selected, with Model (**1**–**6**) representing YOLOv5n, YOLOv8n, YOLOv8-obb, YOLOv11n, YOLOv12n, and OE-YOLO, respectively. The red arrow points to error detection, and the orange arrow points to duplicate detection.

To gain a more intuitive understanding of the improvements of OE-YOLO within the YOLO series, which are more likely to be deployed in real-time edge detection systems, the detection results of various YOLO series models (namely YOLOv5n, YOLOv8n, YOLOv8-obb, YOLOv11n, YOLOv12n and OE-YOLO) on three randomly selected rice panicle images are compared, as shown in Figure 17 [32]. During inference, non-maximum suppression (NMS) employs an IoU threshold of 0.35 to minimize redundant detections while preserving valid neighboring panicles in densely planted fields [33]. In this figure, incorrect detections are indicated by red arrows and duplicate detections are marked with orange arrows.

Upon observing the results, YOLOv5 and YOLOv8 are found to generate a relatively large number of error and duplicate detections. This can be attributed to their incapability of effectively handling the complex shapes and orientations of rice panicles in the field. Their bounding boxes often enclose excessive background regions or fail to accurately localize the rice panicles, resulting in misclassifications and redundant detections. YOLOv8-obb, with its oriented bounding boxes, shows some improvement in conforming to the shapes of rice panicles and reducing background clutter. However, it still has a certain number of error and duplicate detections, suggesting that its feature-extraction and object-localization capabilities could be further enhanced. YOLOv11n and YOLOv12 also exhibit limitations. Despite being advanced object-detection models, it fails to accurately detect some rice panicles and has misclassification and duplicate detection issues due to their convolutional modules’ lack of adaptability to the complex rice-field environment.

In contrast, OE-YOLO demonstrates superior performance. Its bounding boxes closely fit the actual shapes and orientations of rice panicles, minimizing the inclusion of background regions. Significantly fewer error and duplicate detections are observed compared to other models.

## 4. Discussion

### 4.1. Discussion on Backbone Architecture Selection

The integration of EfficientNetV2 as the backbone network in OE-YOLO demonstrates a superior balance between detection accuracy and computational efficiency compared to alternative architectures like EMO, ShuffleNet, ARC, LSKNet, GhostNetV2, MobileNetV3, and MobileNetV4, as evidenced by the comparative metrics in Table 7 [34,35,36,37,38,39,40]. Notably, ARC achieves a competitive mAP50 of 86.1% by integrating adaptive rotation-aware convolutions, yet its computational demand (7.5 GFLOPs) and parameter count (2.68 M) exceed those of EfficientNetV2 (4.8 GFLOPs, 2.45 M). Similarly, GhostNetV2 exhibits suboptimal efficiency, with 7.2 GFLOPs and 6.48 M parameters, despite yielding only 84.4% mAP50. In contrast, EfficientNetV2 attains the highest mAP50 (86.9%) while maintaining a lean parameter count (2.45 M) and moderate computational demand, outperforming lightweight counterparts like MobileNetV3 (83.3% mAP50, 3.8 GFLOPs) in accuracy without significant resource inflation.

This advantage stems from EfficientNetV2’s hybrid scaling strategy, which systematically optimizes network depth, width, and resolution to enhance multi-scale feature extraction—a critical capability for detecting rice panicles across varying growth stages and occlusion patterns. For instance, its high-resolution stem layer preserves fine-grained details of small grains, while fused-MBConv blocks reduce redundancy in shallow layers, enabling efficient propagation of discriminative features to subsequent dynamic convolution modules (C3k2_DConv). The synergy between these components is particularly evident in dense scenarios: EfficientNetV2-based OE-YOLO achieves a reduction in orientation estimation errors compared to ShuffleNet variants, as the backbone’s hierarchical features provide robust spatial priors for OBB-based angle regression.

**Figure 18:**
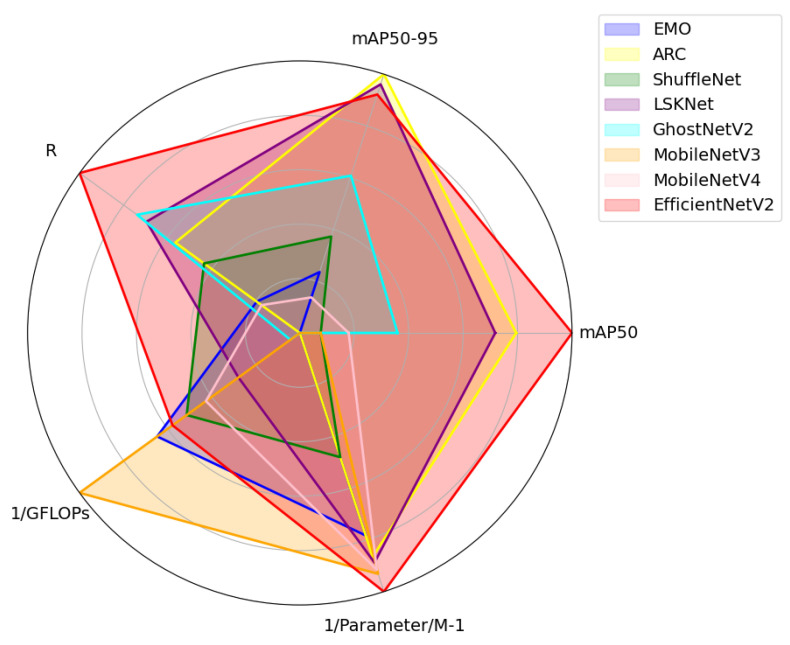
Performance comparison of the eight models.

To compare the performance of each model more intuitively, each metric has been normalized, as shown in Figure 18.

Although replacing the backbone network of YOLOv11 with ARC achieves the highest mAP50-95, it is evident that the performance using EfficientNetV2 is represented as the largest red area in the figure, demonstrating its balance between accuracy and efficiency.

These findings validate EfficientNetV2 as the optimal backbone for OE-YOLO, particularly in agricultural contexts where model deployability and real-time performance are paramount. Its ability to harmonize high accuracy with operational efficiency positions it as a versatile solution for field-scale rice monitoring systems, bridging the gap between academic benchmarks and on-device application requirements.

### 4.2. Visualizing OE-YOLO’s Superiority in Rice Panicle Detection: Insights from Grad-CAM Heatmaps

The heatmaps generated by the Grad-CAM algorithm serve as a powerful tool for visualizing the decision-making process of object-detection models [41]. In these heatmaps, regions with higher intensity values, typically represented by warmer colors such as red and yellow, indicate areas that the model deems more important for its detection decisions. Conversely, regions with lower intensity values, shown in cooler colors like blue, are considered less significant. For the rice panicle detection context, in the heatmaps related to rice images, the high-intensity areas highlight the parts of the rice panicles that the model focuses on when identifying and localizing rice objects.

**Figure 19:**
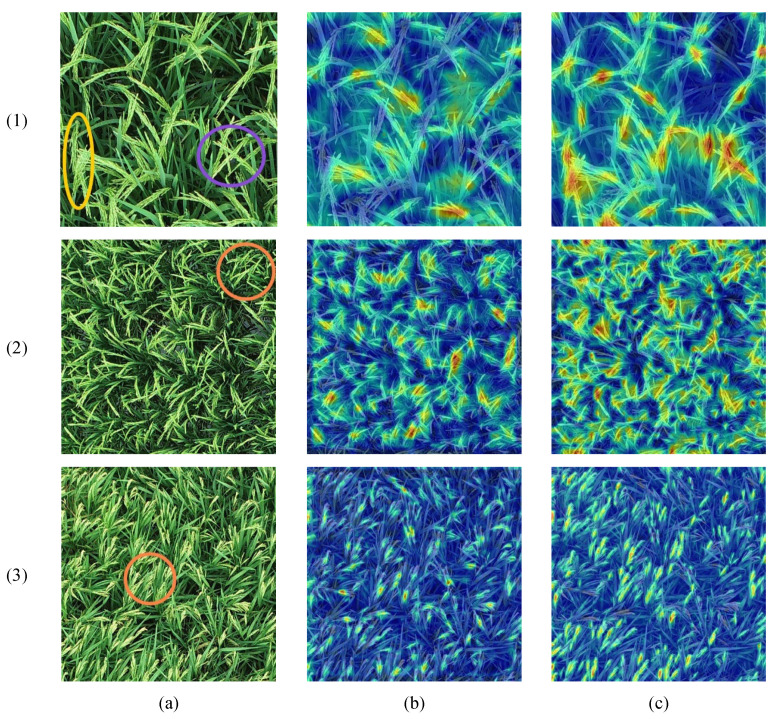
Heatmap visualization results of rice panicles of different sizes. Three images of rice panicles with different sizes are selected, and (**a**–**c**) represent RGB images, YOLOv11, and OE-YOLO, respectively. Purple, yellow, and orange circles indicate intersecting, co-directional, and densely distributed rice panicles that are prone to confusion, respectively.

Figure 19 showcases heatmap visualizations of rice panicles of varying sizes, offering a clear contrast between the results of YOLOv11 and OE-YOLO. In the heatmaps of YOLOv11, regardless of the size of the rice panicles, the distribution of importance appears rather uniform across the rice-related regions. This indicates that the model extracts features in a more generalized fashion, lacking the capacity to effectively differentiate between different levels of significance within the rice panicles.

Turning to the comparison in terms of intersecting, co-directional, and densely distributed rice panicles, as indicated by the purple, yellow, and orange circles in Figure 19, respectively. For intersecting rice panicles marked by the purple circles, YOLOv11 fails to precisely distinguish the individual panicles within the overlapping areas. Its HBB are ill-equipped to conform to the intricate shapes formed by the intersections, leading to a lack of clear separation in the heatmap representation. In contrast, OE-YOLO, leveraging its oriented bounding boxes, can more accurately enclose each intersecting panicle. The C3k2_DConv modules adaptively adjust their convolution operations, enabling the model to better identify the boundaries and features of the intersecting panicles, which is reflected in the more distinct and accurate heatmap patterns.

Regarding co-directional rice panicles indicated by the yellow circles, YOLOv11’s generalized feature-extraction mechanism struggles to differentiate between them due to the lack of prominent orientation differences. It often treats multiple co-directional panicles as a single entity or fails to accurately localize each one, resulting in a blurred heatmap response in these regions. OE-YOLO’s backbone network, EfficientNetV2, endows it with enhanced feature-extraction capabilities. It can capture the fine-grained details and subtle variations among co-directional panicles. Coupled with the adaptability of C3k2_DConv, which can dynamically adjust its receptive fields and filter weights based on the input features, OE-YOLO can more effectively distinguish between co-directional panicles, as evidenced by the more focused and discriminative heatmap regions.

For densely distributed rice panicles denoted by the orange circles, YOLOv11 is prone to producing numerous error and duplicate detections due to its limited ability to handle complex spatial information in such crowded scenarios. The heatmaps of YOLOv11 show a chaotic and less discriminative pattern in these areas. OE-YOLO can better fit the shapes of densely packed panicles and accurately detect and separate individual panicles. The heatmaps of OE-YOLO in these regions exhibit more organized and accurate patterns, indicating its superior performance in handling densely distributed rice panicles.

### 4.3. Application of Rice Panicle Counting

**Figure 20:**
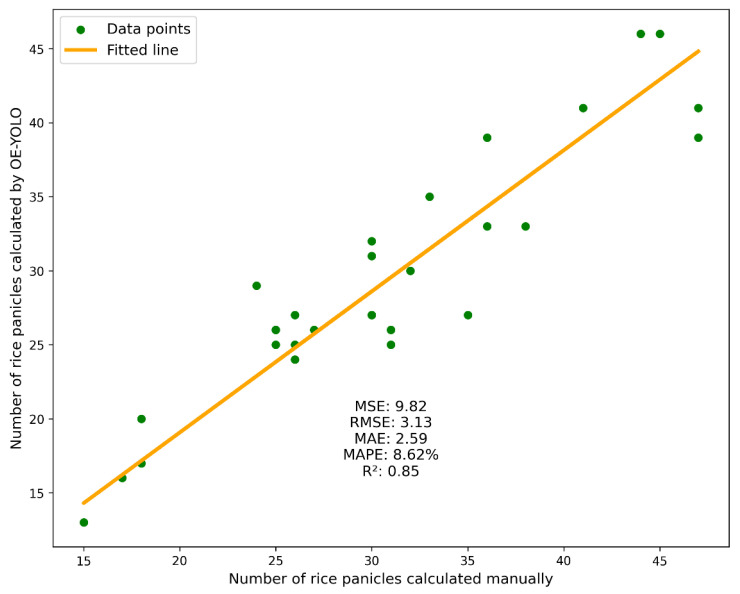
Result of counting the rice panicles.

The feasibility of employing OE-YOLO for rice panicle yield estimation is substantiated by its robust performance in quantifying panicle counts under field conditions. As shown in Figure 20, among the 30 experimental rice panicles randomly selected from EXP1, it can be observed that there is a strong linear correlation between the manually counted values and the estimates obtained from OE-YOLO, with R^2^, MAE, and RMSE being 0.85, 2.59, and 3.13, respectively, and the deviation is limited to a low error threshold. The model’s precision is further evidenced by an 8.62% MAPE (mean absolute percentage error), indicating consistent alignment with ground-truth data even under dense planting configurations.

Notably, the fitted regression line exhibits minimal dispersion across the range of 15–40 panicles per image, confirming OE-YOLO’s scalability for high-density scenarios. Errors predominantly arise in regions with more than 40 panicles, where morphological similarity between adjacent panicles challenges even human annotators. Nevertheless, the 8.62% MAPE underscores the model’s superiority over conventional manual counting methods, which often fail to disentangle intertwined panicles.

This achievement is of great significance for subsequent research on real-time rice yield estimation. By combining the panicle counts detected by OE-YOLO in images captured at different flight heights of the laser-equipped UAVs, a more accurate yield prediction model can be established. The laser data can provide height information from UAV to rice canopy, and when integrated with the panicle count data from different heights, it allows for a more comprehensive understanding of the rice growth situation. The height information obtained by the laser can help correct the panicle count in cases where the density of rice plants varies at different heights. Through data fitting and analysis, a functional relationship between the flight height of the UAV, the number of panicles in the images, and the actual rice yield can be established. This relationship can then be used to estimate the yield of large-scale rice fields in real-time, providing valuable data support for farmers to make scientific decisions regarding irrigation, fertilization, and harvesting.

### 4.4. Broader Impact

The proposed OE-YOLO holds significant implications for democratizing precision agriculture technologies, particularly in resource-limited regions where small-scale farmers constitute the backbone of rice production. By prioritizing computational frugality, the model enables real-time panicle detection on low-cost edge devices such as entry-level UAVs, circumventing the need for high-end computing infrastructure. This accessibility is critical in regions where the area of farmland is limited, and financial constraints restrict investment in advanced agricultural technology. By supporting flexible flight heights, farmers can balance battery efficiency and detection fidelity, using high flight heights for large-area health assessment and low flight heights for yield estimation, which is helpful for directly reducing operational expenditures.

Furthermore, the modular design of OE-YOLO positions it as a versatile tool for crop detection beyond rice. For instance, wheat and barley exhibit similar challenges in field environments such as dense spikelet distributions, variable growth orientations, and occlusion caused by wind or overlapping leaves, all of which align with OE-YOLO’s strengths in handling morphological complexity. The model’s lightweight architecture further ensures compatibility with edge devices commonly used in small-scale farming systems, enabling cost-effective deployment across crops with limited computational resources.

By retraining the backbone on annotated datasets of wheat spikes or barley panicles, OE-YOLO could adapt to species-specific features such as the elongated rachis of wheat or the compact spike clusters of barley. The C3k2_DConv module’s ability to prioritize kernel configurations based on input features would naturally extend to these crops, as their detection requires adaptive attention to scale variations between immature and mature growth stages. This scalability is critical for regions cultivating mixed crops, where farmers often manage intercropped fields with rice, wheat, and legumes simultaneously. Such efforts could establish a unified framework for multi-crop phenotyping, reducing the need for redundant model development while promoting knowledge transfer between crop-specific precision agriculture communities.

### 4.5. Limitations and Future Work

While OE-YOLO demonstrates robust performance in rice panicle detection under controlled conditions, several limitations warrant discussion, some limitations still exist.

First, while OE-YOLO demonstrates robust performance under controlled conditions, its reliability in extreme weather scenarios requires further scrutiny. Heavy rain, for instance, introduces multifaceted challenges: raindrop adhesion to UAV lenses distorts panicle contours through motion blur, while water accumulation on rice leaves amplifies specular reflections that mimic panicle textures in RGB imagery. Such artifacts reduce the discriminative power of edge features critical for OBB-based orientation regression, particularly for slender panicle tips susceptible to occlusion. These effects are exacerbated during the filling stage when dense panicle clusters create overlapping patterns that further confuse feature extraction under low-contrast rainy conditions. A more robust solution involves integrating near-infrared (NIR) sensors to exploit panicle-specific spectral signatures less affected by surface water reflections, though this necessitates UAV hardware modifications beyond the scope of our current RGB-based framework. Future studies could also explore temporal fusion of multi-frame UAV captures to statistically suppress transient rain artifacts, leveraging the inherent persistence of panicle structures against ephemeral weather noise.

Second, the model’s emphasis on small-target detection occasionally fragments branched panicle clusters into multiple independent instances. This arises from the inherent complexity of rice panicle morphology during late growth stages, where secondary branches bifurcate from the main axis at acute angles (15–40°), creating dense, interconnected structures that mimic separate entities in 2D imagery. The slender geometry of these branches further exacerbates feature ambiguity, as convolutional kernels struggle to distinguish branching nodes from background textures. In the future, incorporating depth cues from LiDAR or stereo cameras would enable 3D morphological analysis, distinguishing overlapping branches in planar projections. Such enhancements aim to bridge the gap between pixel-level detection and agronomically meaningful panicle counting, critical for yield prediction accuracy.

Third, the performance decline at elevated UAV flight heights stems primarily from the inverse relationship between spatial resolution and coverage efficiency. While higher flight heights enhance operational throughput by capturing larger field areas, the consequent reduction in ground sample distance blurs critical morphological signatures of rice panicles—particularly their slender axes and branching patterns rendering them visually akin to background elements like soil clods or weed clusters. This resolution-induced ambiguity is exacerbated at filling stage, where overlapping panicles create dense canopies that challenge even human visual interpretation under low-resolution conditions. Future systems could integrate LiDAR-derived canopy height models with RGB imagery, which is a strategy compatible with edge devices where 3D structural data compensates for 2D resolution loss by probabilistically localizing panicles through elevation cues.

Fourth, although OE-YOLO’s Oriented Bounding Boxes (OBB) enhance orientation adaptability, its performance in direct-seeded rice fields—characterized by irregular and random plant distributions—remains untested. This is a critical limitation, as such fields may introduce additional occlusion, overlapping, and morphological variations compared to structured transplanting systems.

Future research will focus on four directions:

(1) Generalizing OE-YOLO’s architecture for cross-crop adaptability via meta-learning techniques, particularly for morphologically similar cereals like wheat and barley;
(2) Implementing edge-device optimization strategies to deploy lightweight variants on agricultural robots for in-field, low-latency monitoring;
(3) Developing laser-augmented UAV platforms to correlate flight altitude with multi-modal sensor data, establishing real-time yield estimation frameworks through adaptive sensor fusion.
(4) Further strengthening the validation of this model, including overcoming existing limitations and improving the detection methods for rice panicles under different weather conditions and flight heights, comparing with more OBB models that can be used for real-time object detection by adjusting the high-accuracy architecture for efficient execution on resource-constrained hardware. Furthermore, developing validation of OE-YOLO on direct-seeded datasets to assess its robustness under unstructured planting patterns, expanding its practical applicability to broader agricultural practices.

These advancements aim to bridge the gap between laboratory-grade accuracy and scalable farm-level deployment, ultimately advancing precision agriculture toward autonomous, data-driven decision-making ecosystems.

## 5. Conclusions

In the realm of rice panicle detection within complex agricultural environments, the OE-YOLO model has been developed to address the challenges posed by the small size, dense distribution, diverse growth directions, and background confusion of rice panicles. The model was optimized through three key modifications to the YOLOv11 framework: the adoption of OBB, the integration of EfficientNetV2 as the backbone network, and the introduction of the dynamic convolution-enhanced C3k2_DConv module.

A series of experiments demonstrated the performance of OE-YOLO. Ablation experiments verified the effectiveness of each improvement component. It was found that OBB alone could increase the mAP50 to 84.7%, EfficientNetV2 improved computational efficiency while maintaining relatively high accuracy (mAP50 of 78.8%), and C3k2_DConv enhanced the feature-extraction ability, leading to a mAP50 of 81.2%. When all improvements were combined, OE-YOLO achieved a mAP50 of 86.9%. Furthermore, it has been verified that the use of EfficientNetV2 and the position of C3k2_DConv demonstrated better performance. The model also demonstrated improvements in mAP50 of 8.3%, 6.9%, 6.7%, and 16.6% compared to YOLOv11 in rice panicle detection at heights of 3 m and 10 m during the heading and filling growth stages, respectively.

The superiority of OE-YOLO has been further validated through comparative experiments. This model outperforms other models in both detection accuracy and computational efficiency, outperforming YOLOv8-obb and YOLOv11 by 2.8% and 8.3%, respectively.

In conclusion, OE-YOLO provides a reliable and efficient solution for rice panicle detection in the field. It was proven to be effective in accurately detecting rice panicles under various conditions, with a relatively low number of parameters (2.45 M) and computational cost (4.8 GFLOPs). The model’s success lies in its ability to leverage the advantages of OBB, EfficientNetV2, and C3k2_DConv. This research contributes to the development of deep-learning-based object-detection methods in agriculture and offers practical value for precision agriculture, enabling more accurate rice yield prediction, and ultimately, more effective agricultural management.

## Figures and Tables

**Table 1:** Original rice panicle dataset.

Experiment	Collection Time	Growth Stage	RGB Images
EXP.1	17 June 2021	Heading	303
EXP.1	28 June 2021	Filling	493
EXP.1	24 June 2024	Heading	133
EXP.1	5 July 2024	Filling	143
EXP.2	18 October 2021	Heading	556
EXP.2	26 October 2021	Filling	557

**Table 2:** Structure of EfficientNetV2.

Stage	Operator	Channels	Stride	Layers
0	Conv	24	2	1
1	Fused-MBConv1	24	1	2
2	Fused-MBConv4	48	2	4
3	Fused-MBConv4	64	2	4
4	MBConv4, SE0.25	128	2	6
5	MBConv6, SE0.25	160	1	9
6	MBConv6, SE0.25	256	2	15
7	Conv and Pooling and FC	1280	-	1

**Table 3:** Ablation experiment.

YOLOv11n	OBB	EfficientNetV2	C3k2_DConv	mAP50/%	mAP50-95/%	Parameter/M	GFLOPs
√				78.6	42.7	2.58	6.3
√	√			84.7	49.8	2.65	6.6
√		√		78.8	49.4	2.09	4.5
√			√	81.2	45.2	2.94	6.2
√	√	√		84.5	49.7	2.15	4.7
√	√	√	√	86.9	50.6	2.45	4.8

**Table 4:** Comparison of detection accuracy and recall rate between YOLOv11 and OE-YOLO under different heights and stages.

		3 m		10 m	
		Heading	Filling	Heading	Filling
P/%	YOLOv11	73.0	59.9	59.1	53.9
	OE-YOLO	83.9	61.8	57.9	63.4
R/%	YOLOv11	72.7	65.2	62.0	56.5
	OE-YOLO	82.8	76.1	75.3	73.0

**Table 5:** Results of placing c3k2_dconv modules in different positions.

Serial Number	C3k2_DConv Location for Different Size Feature Maps			Evaluation Metrics			
	80 × 80	40 × 40	20 × 20	mAP50/%	mAP50-95%	Parameter/M	GFLOPs
1	-	-	-	84.5	49.7	2.15	4.7
2	√			85.5	49.9	2.22	5.0
3		√		84.7	50.0	2.27	5.3
4			√	84.1	49.0	2.43	4.9
5	√	√		85.1	49.6	2.28	4.9
6	√		√	84.9	50.2	2.44	4.9
7		√	√	84.8	50.1	2.49	5.3
8	√	√	√	86.9	50.6	2.45	4.8

**Table 6:** Comparative Experimental Results.

Model	mAP50/%	Parameter/M	GFLOPs
YOLOv5n	75.5	2.28	5.9
YOLOv8n	76.7	2.68	6.8
YOLOv8-obb	84.1	2.76	7.2
YOLOv11n	78.6	2.58	6.3
YOLOv12n	80.0	2.53	5.8
Oriented R-CNN	80.8	41.43	211.4
R3Det_tiny	81.1	37.15	231.9
S^2^A-Net	85.2	38.54	196.2
FCOSR	84.3	31.89	206.2
OE-YOLO	86.9	2.45	4.8

**Table 7:** Results of discussion on Backbone Architecture Selection.

Model	mAP50/%	mAP50-95/%	Parameter/M	GFLOPs
EMO	83.0	47.1	2.84	4.6
ShuffleNet	83.3	47.8	3.62	5.0
ARC	86.1	51.0	2.68	7.5
LSKNet	85.8	50.8	2.64	5.9
GhostNetV2	84.4	49.0	6.48	7.2
MobileNetV3	83.3	45.9	2.56	3.8
MobileNetV4	83.7	46.6	2.59	5.3
EfficientNetV2	86.9	50.6	2.45	4.8

## Data Availability

The datasets in this study are available from the corresponding author on reasonable request.
